# Evaluation of a Cell-Free Collagen Type I-Based Scaffold for Articular Cartilage Regeneration in an Orthotopic Rat Model

**DOI:** 10.3390/ma13102369

**Published:** 2020-05-21

**Authors:** Marta Anna Szychlinska, Giovanna Calabrese, Silvia Ravalli, Anna Dolcimascolo, Paola Castrogiovanni, Claudia Fabbi, Caterina Puglisi, Giovanni Lauretta, Michelino Di Rosa, Alessandro Castorina, Rosalba Parenti, Giuseppe Musumeci

**Affiliations:** 1 Department of Biomedical and Biotechnological Sciences, Anatomy, Histology and Movement Sciences Section, School of Medicine, University of Catania, 95123 Catania, Italy; mszychlinska@unict.it (M.A.S.); silviaravalli@gmail.com (S.R.); pacastro@unict.it (P.C.); giovannilau91@hotmail.it (G.L.); mdirosa@unict.it (M.D.R.); 2 Department of Biomedical and Biotechnological Sciences, Physiology Section, School of Medicine, University of Catania, 95123 Catania, Italy; soniacalabrese@hotmail.com (G.C.); anna.dol@alice.it (A.D.); parenti@unict.it (R.P.); 3 Fin-Ceramica Faenza, 48018 Faenza, Italy; claudia.fabbi@finceramica.it; 4 Istituto Oncologico del Mediterraneo (IOM), 95029 Viagrande, 95123 Catania, Italy; caterinapuglisi.cp@gmail.com; 5 School of Life Science, Faculty of Science, University of Technology Sydney, Sydney, NSW 123, Australia; Alessandro.Castorina@uts.edu.au; 6 Discipline of Anatomy & Histology, School of Medical Sciences, The University of Sydney, Sydney, NSW 123, Australia; 7 Research Center on Motor Activities (CRAM), University of Catania, 95123 Catania, Italy; 8 Department of Biology, Sbarro Institute for Cancer Research and Molecular Medicine, College of Science and Technology, Temple University, Philadelphia, PA 19122, USA

**Keywords:** articular cartilage lesion, cartilage regeneration, cartilage tissue engineering, collagen I-based scaffold, orthotopic implantation

## Abstract

The management of chondral defects represents a big challenge because of the limited self-healing capacity of cartilage. Many approaches in this field obtained partial satisfactory results. Cartilage tissue engineering, combining innovative scaffolds and stem cells from different sources, emerges as a promising strategy for cartilage regeneration. The aim of this study was to evaluate the capability of a cell-free collagen I-based scaffold to promote cartilaginous repair after orthotopic implantation in vivo. Articular cartilage lesions (ACL) were created at the femoropatellar groove in rat knees and cell free collagen I-based scaffolds (S) were then implanted into right knee defect for the ACL-S group. No scaffold was implanted for the ACL group. At 4-, 8- and 16-weeks post-transplantation, degrees of cartilage repair were evaluated by morphological, histochemical and gene expression analyses. Histological analysis shows the formation of fibrous tissue, at 4-weeks replaced by a tissue resembling the calcified one at 16-weeks in the ACL group. In the ACL-S group, progressive replacement of the scaffold with the newly formed cartilage-like tissue is shown, as confirmed by Alcian Blue staining. Immunohistochemical and quantitative real-time PCR (qRT-PCR) analyses display the expression of typical cartilage markers, such as collagen type I and II (*ColI* and *ColII*), *Aggrecan* and *Sox9*. The results of this study display that the collagen I-based scaffold is highly biocompatible and able to recruit host cells from the surrounding joint tissues to promote cartilaginous repair of articular defects, suggesting its use as a potential approach for cartilage tissue regeneration.

## 1. Introduction

The repair and healing capacity of articular cartilage after injury is complicated due to its avascular, hypo-cellular and aneural nature [1]. For this reason, even a minor lesion may lead to progressive damage and cartilage degeneration, determining osteoarthritis (OA) development. OA is a common progressively degenerative disease involving primarily articular cartilage and further all joint tissues and leading to severe pain and joint disability [2,3]. Several therapeutic approaches have been developed for the treatment of articular cartilage defects, including autografts and osteochondral allografts, microfracture, autologous chondrocyte and mesenchymal stem cell-based therapies [4,5]. However, the results reported in this field showed limited satisfactory results and the management of chondral defects remains a big challenge. This is mostly due to the biochemical and mechanical properties of the obtained engineered cartilage, which often do not match those of the native tissue [6].

Tissue engineering for regenerative approaches emerges as one of the most promising biomedical applications for cartilage tissue regeneration. The latter is based on the use of innovative biomaterials, which act as scaffolds, mimicking a three-dimensional (3D) extracellular matrix (ECM) microenvironment, with or without the use of chondrocytes or mesenchymal stem cells from different sources [7,8,9,10]. Over the past decades, several advances in this field have arisen, based on the innovative techniques used for biomaterial characterization, design and functionalization [11,12].

The biomaterials used in cartilage engineering approaches should provide mechanical support, shape, and cell-scale architecture for neo-tissue formation as cells expand and organize. In addition to defining the 3D architecture for the neo-tissue, the scaffold provides the microenvironment (synthetic temporary ECM) for regenerative cell recruitment, support, proliferation, differentiation and, finally, neo-tissue formation [13]. The most commonly used degradable biomaterials nowadays include some synthetic polyesters such as poly(l-glycolic acid) (PLGA) and poly(l-lactic acid) (PLLA) and natural biopolymers such as collagen, alginate, fibrin and chitosan [14,15]. The natural polymers show better biological properties that are more suitable for the native cartilage microenvironment, promoting required biocompatibility, cellular responses and biodegradability [16]. Among them, collagen type I is widely used for scaffold construction and cartilage tissue engineering approaches, due to its high biocompatibility and widespread clinical usage [17,18,19,20]. Although there are many scaffolds based on collagen, its long-term performance still shows an inferior mechanical property and limited chondrogenic capacity. For this reason, improvements of the physical and structural properties of collagen I-based scaffolds are still required, as stated and highlighted by Irawan et al., in the recently published review [21]. Recently, the biocompatibility and the chondrogenic potential of a new 3D collagen type I-based scaffold has been evaluated by our research group both in vitro and in vivo (etherotopic implantation) [22,23]. Our in vitro results performed using this scaffold in combination with human adipose-tissue derived mesenchymal stem cells (hADMSCs) showed that the scaffold is able to promote the early stages of chondrogenic cell differentiation and that the addiction of specific inductive factors induces complete differentiation as highlighted both by specific cartilage markers expression and typical chondrocyte morphology [22]. The most important cartilage markers are represented by glycosaminoglycans (GAGs), collagen type II and aggrecan, which are typical ECM components of hyaline cartilage that characterise the articular cartilage of diarthrodial joints. Collagen type I, instead represents the main constituent of the fibrocartilage matrix, which possesses completely different mechanical properties compared to hyaline cartilage [1,6]. Another important chondrogenic marker is represented by SOX9, a transcription factor expressed by chondrocytes, which are the only cell type present within the cartilage tissue. It has been identified as a regulator of the chondrocyte lineage, essential for chondrocyte differentiation and cartilage formation. It is associated with the enhancement of collagen II and aggrecan synthesis within the cartilage matrix [24]. In vivo data obtained by subcutaneous implantation (heterotopic model) of a cell free scaffold showed that it is biocompatible and able to recruit host cells and to guide them towards the chondrogenic differentiation [22]. Furthermore, the cartilage-like microstructural properties of this biomaterial, in terms of density and elasticity, were combined in a simple production process [25], which makes this scaffold even very interesting to be evaluated for cartilage regenerative approaches. For this reason, to further evaluate and emphasize the data obtained in our previous studies, the aim of the present work was to continue the validation of this cell-free collagen I-based scaffold in an articular cartilage lesion (ACL) orthotopic model in terms of host cells recruitment, ECM deposition and cartilaginous repair promotion. The results of the present study complete the multi-step evaluation process of the 3D collagen I-based scaffold and may pave the way to the use of the latter in the cartilage regeneration approaches.

## 2. Results

### 2.1. 3D Scaffold Characterization before Implantation

**Figure 1:**
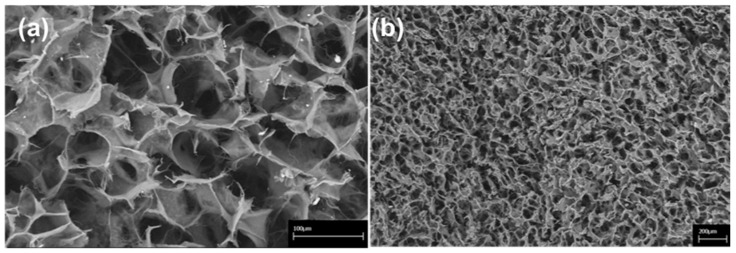
Scanning Electron Microscopy (SEM) images of the collagen-based scaffold. At higher magnification, interconnected collagen fibres are detectable within the scaffold. Scale bars: 100 μm in (**a**); 200 μm in (**b**).

The microstructural and morphological properties of the 3D ColI-based scaffold were evaluated by SEM analysis as previously indicated [22,23]. Briefly, Figure 1 shows SEM images of the 3D scaffold at different magnifications, displaying a high porosity of the scaffold with 3D intersected pores without any defined alignment of the collagen fibers. Pore distribution analysis indicated a frequency ( > 65%) of pores between 40 and 100 μm in size. The swelling test was performed to assess the change of material structure clearly demonstrating that the collagen I-based scaffold is highly hydrophilic and reaches a steady-state in less than 1 min. The volume of absorbed PBS was quantified to evaluate the capability of the collagen scaffold to maintain the liquid assigned to the support of the 3D structure. After swelling, both diameter (6 ± 1%) and thickness (27 ± 9%) of the scaffold appeared significantly increased.

### 2.2. Morphological Evaluation of Explanted Femurs

**Figure 2:**
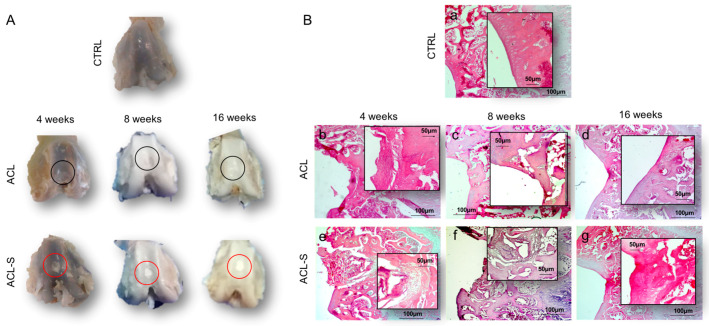
Cartilage repair evaluation through macroscopic and microscopic evaluation. (**A**) Macroscopic evaluation of repair capacity of femoral articular cartilage explants after defect creation indicated with black circles (ACL group) and in vivo scaffold implantation indicated with red circles (ACL-S group) at 4-, 8- and 16-weeks; (**B**) Histological evaluation by H&E staining of femoral articular cartilage samples after defect creation (ACL group) and in vivo scaffold implantation (ACL-S group) at 4-, 8- and 16-weeks: (**a**) control sample presenting a normal cartilage cytoarchitecture; (**b**) ACL group sample at 4-weeks presenting fibrocartilage formation at the defect area level; (**c,d**) ACL group sample at 8- and 16-weeks presenting cartilage calcification corresponding to the defect area level; (**e**) ACL-S group sample at 4-weeks presenting a prechondrogenic mesenchyme-like tissue features at the interface between scaffold and the peri-native tissue; (**f**) ACL-S group sample at 8-weeks presenting matrix deposition within the scaffold, suggesting host cell recruitment and their chondrogenic differentiation; (**g**) ACL-S group sample at 16-weeks presenting a total scaffold reabsorption and replacement with a newly formed cartilage-like tissue. Scale bar: 100 μm. The inserts represent the image magnifications (scale bar: 50 μm) to evidence the morphology changes observed in a time-dependent manner.

**Figure 3:**
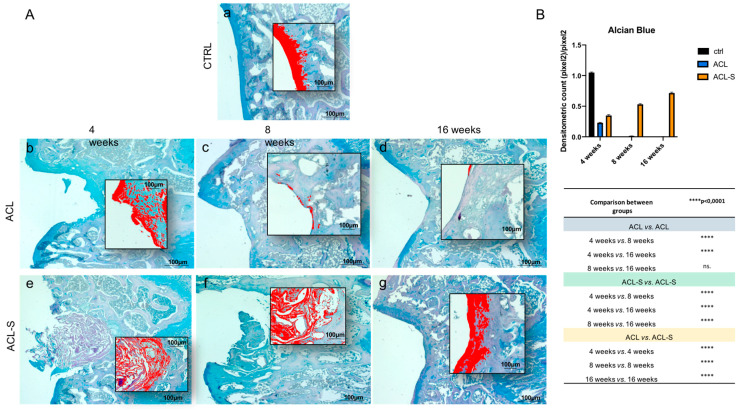
Histochemical evaluation of the deposition of sGAGs in femoral articular cartilage samples at 4-, 8- and 16-weeks post-surgery revealed by the intensity of Alcian Blue staining through computerised densitometric measurements and image analysis. (**A**) The inserts represent the image magnifications (scale bar: 100 μm) analysed by the software: the red colour corresponds to high intensity Alcian Blue staining. (**a**) control sample of articular cartilage; (**b–d**) ACL group samples at 4-, 8- and 16-weeks; (**e–g**) ACL-S group samples at 4-, 8- and 16-weeks. Scale bars: 100 μm. (**B**) Graph representing staining level expressed as densitometric count (pixel^2^) normalized to the area of each section expressed in pixel^2^. Results are presented as the mean ± SD. Two-way ANOVA test followed by Tukey’s multiple comparison test reported that all pairwise comparisons were significantly different (*p*-value < 0.0001) except for ACL 8-weeks vs. ACL 16-weeks, which was not significant (ns.).

To assess the capability of the collagen I-based scaffold to promote cartilage restoration we performed macroscopic (Figure 2A) and microscopic (Figure 2B) evaluation on the explanted femurs at 4-, 8-, and 16-weeks post-surgery and orthotopic implantation. The hematoxylin and eosin (H&E) staining was used to study the microscopic morphology of the femoral articular cartilage in both groups (femurs with implanted collagen I-scaffolds: ACL-S group, and femurs without scaffolds: ACL group) in order to detect alterations. In the control group, articular cartilage showed a normal cytoarchitecture. In the superficial zone, cells appeared flat and small; in the middle and deep zone, chondrocytes were organised in columns; the tidemark was very strong and evident (Figure 2Ba). In the articular cartilage of the ACL group, the general tissue organization was completely altered due to the defect induction. The superficial, middle and deep zones, as well as the tidemark, were not observable anymore at all the time points (Figure 2Bb–d). At 4 weeks post-surgery, the H&E staining revealed a newly formed fibrous tissue (scar tissue) in the superficial zone at the surface of the subchondral bone, corresponding to the defect repair (Figure 2Bb). At 8- and 16-weeks the scar tissue tended to be progressively replaced by a tissue that appeared to be calcified, suggested by the morphological aspect of the tissue and poor proteoglycans deposit, further evidenced by Alcian Blue staining (Figure 3). The peri-native cartilage features appeared totally altered (Figure 2Bc,d). In the articular cartilage of the implanted group (ACL-S), at 4-weeks post-surgery, the H&E staining showed the presence of newly formed tissue at the interface between the subchondral bone and collagen scaffold, which presented morphological features resembling a prechondrogenic mesenchymal-like tissue, characterised by the spindle-shaped cells growing without any apparent internal organisation (Figure 2Be). However, this observation has not been validated by specific stainings and would need to be confirmed. Afterwards, the samples revealed the capacity of the biomaterial to recruit host cells that infiltrated, adhered and grown within the scaffold (8-weeks, Figure 2Bf). A progressive integration and replacement of the degradable collagen scaffold with the reparative newly formed cartilage-like tissue were shown (16-weeks, Figure 2Bg).

The evaluation of cartilage repair was also assessed by the deposition of sulfated glycosaminoglycans (sGAGs) revealed by the intensity of Alcian Blue staining (Figure 3). In the ACL group at 4-weeks, the newly formed fibrous tissue showed a low-intensity blue staining (Figure 3Ab), which diminished progressively and in a significant way with the supposed calcification of the cartilage tissue through the time points and, especially, at 16-weeks (*p*-value < 0.0001, Figure 3Ad,B). In the ACL-S group (Figure 3Ae–g), the Alcian Blue staining was much stronger than in the defect control group (ACL group). The internal repair integrity was underlined by the higher ECM deposition within the scaffolds due to the recruitment of host cells, moreover, it appeared progressive through the time points, especially at 16-weeks post-implantation (*p*-value < 0.0001, Figure 3Af,B).

### 2.3. Ex Vivo Evaluation of Cartilage Regeneration

**Figure 4:**
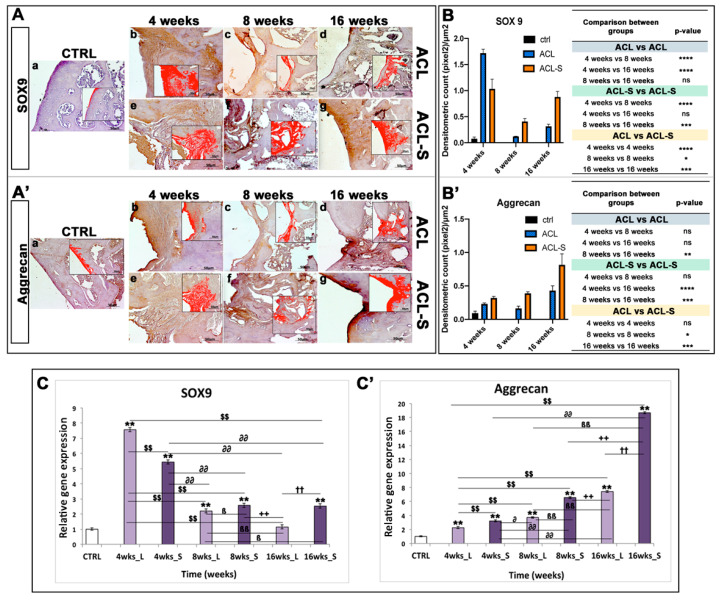
Sox9 and aggrecan evaluation in femoral articular cartilage samples at 4-, 8- and 16-weeks post-surgery. (**A–A’**) Immunohistochemical analyses: (**a**) control sample of articular cartilage; (**b–d**) ACL group samples at 4-, 8- and 16-weeks; (**e–g**) ACL-S group samples at 4-, 8- and 16-weeks. In the inserts, the red colour corresponds to brown staining (immune complexes labelled with chromogen); scale bars 50 μm. (**B–B’**) Graph representing staining level expressed as densitometric count (pixel^2^) normalized to the area of each section expressed in μm^2^. (**C–C’**) Relative quantitation (RQ) of gene expression showing the time-course of *Sox9* and *Aggrecan* in ACL (L) and ACL-S (S) groups, after 4-, 8-, and 16-weeks from surgery. *TUBB4a* has been used as endogenous controls. Results are presented as the mean ± SD. Differences between groups were evaluated by using a two-way ANOVA followed by Tukey’s multiple comparison post-hoc test (*p < 0.05; **p < 0.01; ***p < 0.001; ****p < 0.0001; ns, not significant). * CTRL vs 4-, 8-, 16- wks_L and 4-, 8-, 16-wks_S; $ 4-wks_L vs 8-, 16-wks_L and 4-, 8-, 16-wks_S; ∂ 4-wks_S vs 8-, 16-wks_L and 8-, 16-wks_S; β 8-wks_L vs 16-wks_L and 8-, 16-wks_S; + 8-wks_S vs 16-wks_L and 16-wks_S; † 16-wks_L vs 16-wks_S.

**Figure 5:**
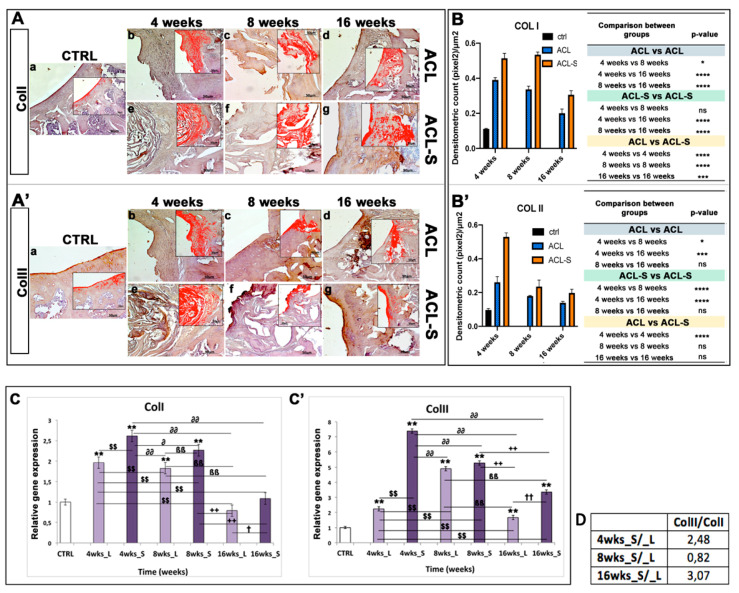
Collagen type I and collagen type II evaluation in femoral articular cartilage samples at 4-, 8- and 16-weeks post-surgery. (**A–A’**) Immunohistochemical analyses: (**a**) control sample of articular cartilage; (**b–d**) ACL group samples at 4-, 8- and 16-weeks; (**e–g**) ACL-S group samples at 4-, 8- and 16-weeks. In the inserts, the red colour corresponds to brown staining (immune complexes labelled with chromogen); scale bars 50 μm. (**B–B’**) Graph representing staining level expressed as densitometric count (pixel^2^) normalized to the area of each section expressed in μm^2^. (**C–C’**) Relative quantitation (RQ) of gene expression showing the time-course of *ColI* and *ColII* in ACL (L) and ACL-S (S) groups, after 4-, 8-, and 16-weeks from surgery. *TUBB4a* has been used as endogenous controls. Results are presented as the mean ± SD. Differences between groups were evaluated by using a two-way ANOVA followed by Tukey’s multiple comparison post-hoc test (*p < 0.05; **p < 0.01; ***p < 0.001; ****p < 0.0001; ns, not significant). (**D**) Table showing the ratio of collagen II/collagen I (ColII/ColI). * CTRL vs 4-, 8-, 16-wks_L and 4-, 8-, 16-wks_S; $ 4-wks_L vs 8-, 16-wks_L and 4-, 8-, 16-wks_S; ∂ 4-wks_S vs 8-, 16-wks_L and 8-, 16-wks_S; β 8-wks_L vs 16-wks_L and 8-, 16-wks_S; + 8-wks_S vs 16-wks_L and 16-wks_S; † 16-wks_L vs 16-wks_S.

Immunohistochemical staining with statistical analysis was carried out in all groups to evaluate cartilage repair through the expression level of SOX9 (Figure 4A), a pivotal transcription factor for cartilage formation and ECM cartilaginous structural molecules, Aggrecan (Figure 4A’), Collagen type I (Figure 5A), and Collagen type II (Figure 5A’).

A very strong expression of SOX9 was seen at 4-weeks post-surgery, especially in the ACL group (Figure 4Ab). It decreased significantly at 8-weeks in both ACL (*p*-value < 0.0001, Figure 4Ac,B) and ACL-S (*p*-value < 0.0001, Figure 4Af,B) groups, to increase again at 16-weeks especially in ACL-S group (*p*-value < 0.001, Figure 4Ag,B). Overall, the SOX9 expression was significantly higher in ACL-S group when compared to defect control group (ACL group), both at 8- (*p*-value < 0.05, Figure 4Af,B) and 16-weeks (*p*-value < 0.0001, Figure 4Ag,B).

The expression profile of aggrecan showed a progressive increase through the time points, with the highest peak at 16-weeks in the ACL-S group (Figure 4A’g). Overall, aggrecan expression was always higher in the ACL-S group when compared to the defect control group and it was significant at 8- (*p*-value < 0.05, Figure 4A’f,B’) and 16-weeks (*p*-value < 0.001, Figure 4A’g,B’).

Collagen type I expression was strong at 4-weeks (Figure 5Ae) and 8-weeks (Figure 5Af), in the ACL-S group, decreasing significantly at 16-weeks post-surgery (*p*-value < 0.0001, Figure 5Ag,B). However, the collagen I expression resulted significantly higher in the ACL-S group when compared to the defect control (ACL group) at all time points: 4-weeks (*p*-value < 0.0001, Figure 5Ae,B), 8-weeks (*p*-value < 0.0001, Figure 5Af,B), 16-weeks (*p*-value < 0.001, Figure 5Ag,B).

A very strong expression of collagen type II was seen at 4-weeks post-surgery, especially in the ACL-S group in which it was significantly higher when compared to the defect control group (ACL group) (*p*-value < 0.0001, Figure 5A’e,B’) and it decreased progressively through the time points (Figure 5A’f,g). The expression profile of collagen II demonstrated a significant difference between the ACL and ACL-S groups at 4-weeks (*p*-value < 0.0001, Figure 5A’b,e,B’) but not at 8- and 16-weeks (*p*-value < 0.05, Figure 5A’c,d,f,g,B’).

A qRT-PCR analysis was performed on total RNA isolated from explanted scaffolds to evaluate the expression of specific genes correlated to cartilage phenotype. The expression profiles of cartilaginous genes, including *ColI*, *ColII*, *Aggrecan* and *Sox9* at 4-, 8-, and 16-weeks were compared to control mRNA levels. Logarithmic RQ values are reported in Figure 4 and Figure 5.

*Sox9* displayed a characteristic peak of expression at 4-weeks both in the ACL (RQ = 7.563) and in ACL-S groups (RQ = 5.432) that decreased progressively over time (ACL group: 8-weeks, RQ = 2.199 and 16-weeks RQ = 1.143; ACL-S group: 8-weeks RQ = 2.580, 16-weeks RQ = 2.528), even if the ACL-S group maintained a higher expression of *Sox9* when compared to the ACL group (Figure 4C).

*Aggrecan* exhibited a lower expression until the 8^th^ week with a peak of expression at the 16^th^ week in both groups, although the ACL-S group (RQ = 18.687) displayed a more pronounced expression than the ACL group (RQ = 7.433) (Figure 4C’).

*ColI* showed an increased expression during the fourth week in both groups, RQ = 1.965 in the ACL group and RQ = 2.612 in the ACL-S group, that decreased down to 0.792 and 1.085 at 16-weeks, respectively for the ACL and ACL-S groups (Figure 5C).

Finally, *ColII* showed a distinctive peak of expression at 4-weeks for ACL-S (RQ = 7.388) with a subsequent decrease and reduced modulation (3.355 orders of magnitude) until week 16; in contrast, the ACL group showed a higher *ColII* expression at 8-weeks (RQ = 4.905) that subsequently decreased to 1.671 orders of magnitude at week 16 (Figure 5C’). Moreover, the gene expression profile data showed a ratio of *ColII/ColI* for ACL-S/ACL groups of 2.48 fold at 4-weeks, 0.82 at 8-weeks and 3.07 at 16-weeks post-surgery (Figure 5D).

## 3. Discussion

The aim of the present study was to evaluate the extent to which the cell-free collagen I-based 3D scaffold might support hyaline cartilage repair of femoral articular cartilage defects, created to reproduce the ACL model, at 16-weeks post-surgery. The concept of using cell-free scaffolds in tissue engineering is widely accepted and has been advanced by Omori et al., in 2008, in an interesting study on laryngeal cartilage reconstruction in a canine model [26], where the authors suggested the successful cartilage reconstruction by the in situ tissue engineering approach.

In the present study, the collagen I-based scaffolds were confirmed to be biocompatible, as already demonstrated in our previous study [23] and as evidenced by the histological analysis of the present study, showing total biodegradation and replacement of the biomaterial with the newly formed cartilage-like tissue at 16-weeks post-implantation (Figure 2B). Moreover, as previously revealed [22,27,28], the scaffolds showed good immune tolerance by the animals, as suggested by the absence of scar-like tissue formation and inflammatory cell infiltration at the interface between the scaffold and peri-native cartilage tissue (Figure 2B). Furthermore, the H&E and Alcian Blue staining demonstrated that the collagen-based scaffold allowed the formation of an articular cartilage-like tissue corresponding to the defect area at the femoropatellar groove level. It was underlined by the significantly higher deposition of sGAGs at 16-weeks post-implantation (Figure 3g) when compared to the defect control group (Figure 3d). The latter showed, instead, newly formed tissue resembling calcified tissue in the area corresponding to the defect, at the same time point. These data were confirmed by immunohistochemical analysis, which showed a higher expression of cartilage markers in ACL-S group samples, when compared to the ACL group samples (Figure 4 and Figure 5), at all the time points. The only exception regards the SOX9 expression, which at its highest peak, corresponded to the ACL group sample at 4-weeks post-surgery (Figure 4Ab). The latter was probably due to the fact that, at 4-weeks, we expected that the formation of fibrous tissue might be preceded by mesenchymal tissue formation, characterized by a high SOX9 expression [29,30]. Afterwards, along with the probably observed calcification-unlike process (8- and 16-weeks post-surgery) in the ACL group, a significant decrease of SOX9 expression was observed (Figure 4Ac,d,B). It probably happened because the recruited host cells were not supported by any 3D structure, like that given by the collagen I-based scaffold (ACL-S group, Figure 4Ae–g). Indeed, it has been widely shown that the 3D architectural support enhances and improves the cartilaginous matrix formation and stability [31,32,33]. SOX9 is a transcription factor that plays a key role in chondrogenesis, both by driving the collagen type II and aggrecan expression and by supporting the survival of chondrocyte [29,34]. Apart from the exception of week 4, the results of immunohistochemistry demonstrated that SOX9 expression was maintained always higher in the ACL-S group, especially at 16-weeks post-implantation. These results were observed also in the expression profiles of collagen II (Figure 5) and, especially, of aggrecan, which the highest peak corresponded to the the ACL-S group at 16-weeks (Figure 4A’g). Another important observation regards the expression profiles of collagen type I and II, which the highest peaks corresponded to the ACL-S group at week 4, as seen in Figure 5Ae,A’e. This was probably due to the fact that at this time point, the collagen I-based scaffolds still conserved their integrity, and have not yet undergone the biodegradation process, which was observable at 8- and, even more, at 16-weeks after implantation (Figure 2 and Figure 3). However, the scaffold has not been specifically labelled and this observation would need to be further confirmed. The collagen II high expression was also justified by the infiltrated cells within the scaffolds, already synthesising this cartilage marker, and occupying a bigger area when compared to the cartilage repair tissue at 8- and 16-weeks (Figure 2). The immunohistochemistry results were further confirmed by the gene expression analysis, which presented the same expression profiles of all the chondrogenic markers, as seen in Figure 4C and Figure 5C. Overall, the results of histological, histochemical, immunohistochemical and gene expression analysis confirmed that implantation of collagen I-based scaffold within the cartilage defects of rats, improved the cartilage tissue regeneration when compared to the group without the scaffolds.

## 4. Materials and Methods

### 4.1. Scaffold Features

Collagen I-based scaffolds used in the present study were manufactured by Fin-Ceramica Faenza SpA (Faenza, Italy). The characterization and process of manufacturing were already widely defined in our previous study [22] and summarised below. These supports present a cylindrical form (8 mm diameter and 5 mm height) and are made up of equine type I collagen gel (1wt%) furnished in aqueous acetic buffer solution (pH = 3.5) (Opocrin SpA, Modena, Italy). The development process and physical and chemical features have been explained previously [22,23]. In Brief, collagen gel was diluted in water and supplied by 0.1 M NaOH solution, up to the isoelectric point (pH = 5.5), in which it precipitated in fibres. Subsequently, it was crosslinked at 37 °C by 48 h long immersion of the fibres in NaHCO_3_/Na_2_CO_3_ (Sigma Aldrich, Milan, Italy) and Merck Millipore, aqueous solution with a 1,4-butanediol-diglycidyl-ether (BDDGE) and freeze-dried for 25 h under vacuum conditions (P = 0.29 mbar) to obtain a porous 3D structure. Finally, the collagen constructs were treated with gamma-rays at a minimum of 25 kGy.

The characterisation of 3D collagen scaffolds from a morphological and microstructural point of view was performed by scanning electron microscopy (SEM) by using an SEM-LEO 438 VP (Carl Zeiss AG, Oberkochen, Germany). Before the analysis, 3D scaffolds were sputter-coated with gold. SEM images were assessed by image J software by calculating the mean pore diameter (mean value of 67 ± 31 microns on a total of 327 pores) (Figure 1). The swelling ability of the material was estimated on 20 cartilaginous cylindrical constructs (ø = 10 mm, h = 4 mm) and determined by evaluating the weight increase and the percent increase in both dimensions [35], as already reported in our previous study [22]. The data analysis did not include the outlier values (Huber test). The porosity and density of collagen constructs were assessed with a glass pycnometer full of highly purified water on n. 20 scaffolds (d = 18 mm; h = 4 30mm) [36]. Pore diameter was then calculated using the geometric volume of the scaffolds and the mean value of the achieved densities [22].

### 4.2. Breeding and Housing of Animals, Experimental Design and Surgery Procedure

Twenty-seven 2-month-old healthy female Wistar outbred rats (Charles River Laboratories, Milan, Italy), with a bodyweight of 300  ± 20 g, were used in the present study. The animals were kept in polycarbonate cages (10.25″W × 18.75″D × 8″H) at controlled humidity and temperature (20–23 °C) throughout the whole period of the experiment, with free access to food and water and a 12 h light/dark photoperiod. The 27 animals were divided into three groups at three different time points as shown in Table 1. The ACL groups consisted of rats submitted to surgical treatment to create defects inducing the ACL model. In the CTRL group, only 4-week samples were taken into account for the analysis.

**Figure 6:**
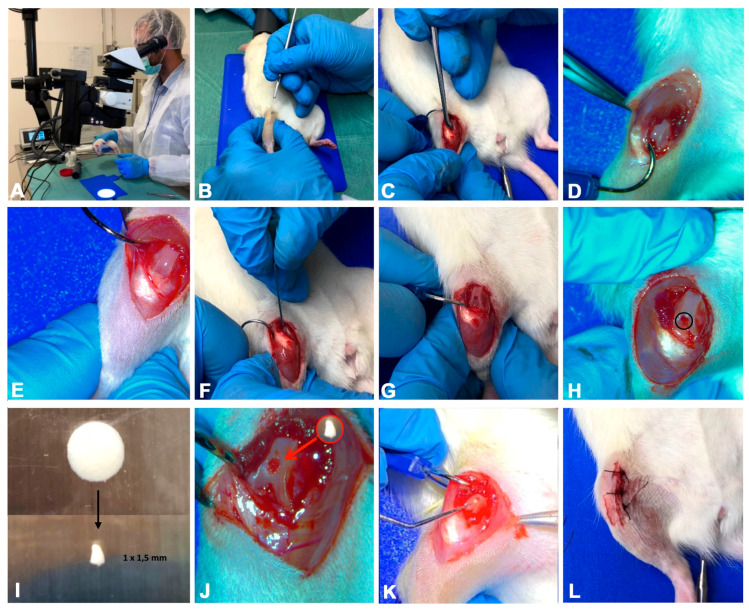
The photographs representing the surgical procedure performed to create defects (ACL induction) and to implant a collagen I-based scaffold. (**A**) total anaesthesia induction; (**B**) knee joint preparation for the incision; (**C–E**) vertical incision through the skin and articular capsule along the medial border and lateral displacement of the patella; (**F–H**) hole formation at the level of the femoropatellar groove in both ACL and ACL-S groups; (**I**) collagen I-based scaffold preparation and cutting; (**J**) collagen I-based scaffold implantation into the hole in the ACL-S group only; (**K**,**L**) patella replacement and the articular capsule and skin suture.

Total anaesthesia (30 mg/kg Zoletil 100 + altadol 5 mg/kg + maintenance mixture of O_2_ and isoflurane 2%–2.5%, Vibrac, Milan, Italy) was used for the surgery procedure. The electric clipper was used to shave the right limb anterior portion, which was then cleaned with povidone iodine (Sceptre Medical, New Delhi, India). The vertical incision was made through the medial border of the skin around the knee cap and, subsequently, through the articular capsule. Afterwards, the patella was moved laterally to expose the right limb femorotibial joint. By flexing the knee the femoral condyles were exposed and a 1 mm × 1.5 mm sharp surgical forceps and needles were used to make a hole within the articular cartilage at the level of the femoropatellar groove of the right limb. Each rat from the ACL-S group received the same treatment, i.e., the collagen scaffolds were sterilely cut into 1 mm × 1.5 mm pieces and implanted into the femoral condyle hole on the right leg while no material was implanted in rats from the ACL group. The implantation was made using press-fit fixation, without supplementary fixation devices. The patella was then removed back, and the articular capsule and skin were sutured by using a 3–0 polydioxanone suture (Figure 6). Post-surgery, one dose of antibiotic Convenia^®^ 0.1 mL/kg, (Vibrac, Milan, Italy), anti-inflammatory (Meloxicam 1 mg/kg) and analgesic (Tramadol 5 mg/kg) drugs, was administered for 3 days. After surgery, the animals were free to move in the cages without joint immobilization. During all the experimental period the suffering of animals was monitored through their observation (weight, lameness, fur appearance, consumption of food and water), performed once a day. The animals from all groups (CTRL, ACL and ACL-S) and all the sub-groups (4-, 8- and 16-weeks) after the surgical procedures were sacrificed by carbon dioxide (CO_2_) overdose. After euthanasia, femurs were explanted, cleaned of soft tissues and the samples were processed for histological, immunohistochemical and gene expression analyses. All the procedures were carried out at the Center for Advanced Preclinical In Vivo Research (CAPIR), University of Catania, conformed to the guidelines of the Institutional Animal Care and Use Committee (I.A.C.U.C.) of the University of Catania (Protocol n. 2112015-PR of the 14.01.2015, Italian Ministry of Health). The experiments were conducted in accordance with the Italian Animal Protection Law (116/1992) and the European Community Council Directive (86/609/EEC).

### 4.3. Histology Analysis

Cartilage samples were washed in phosphate-buffered saline (PBS, Bio-Optica, Milano, Italy), fixed in 10% buffered-formalin (Bio-Optica, Milan, Italy) for 24 h at room temperature. Afterwards, the samples were dehydrated in graded ethanol (Bio-Optica, Milan, Italy), cleaned in xylene (Bio-Optica, Milan, Italy) and paraffin-embedded (Bio-Optica, Milan, Italy), being careful to preserve the desired anatomical orientation. For the general evaluation of the morphological structure of the cartilage, the slides of 4–5 µm thickness were cut from the obtained paraffin blocks and haematoxylin and eosin-stained (H&E; Bio-Optica, Milan, Italy) as previously described [33]. The samples were then examined with a Zeiss Axioplan light microscope (Carl Zeiss, Oberkochen, Germany) and by a digital camera (AxioCam MRc5, Carl Zeiss), used to take images.

For qualitative histological analysis the following parameters were analysed:

- The type of repaired tissue on the lesion surface (cartilaginous, fibrous or calcified);
- Capability of the collagen I-based scaffold to recruit host cells and promote cartilaginous matrix deposition;
- The scaffold biocompatibility and reabsorption of the collagen I-based scaffold.

### 4.4. Analysis of sGAGs by Histochemistry

The samples were obtained as described above. Alcian Blue staining (Bio-Optica, Milan, Italy) was used to evaluate the expression of glycosaminoglycans (GAGs). The evaluation was made by computerised densitometric measurements. The samples were observed with a Zeiss Axioplan light microscope (Carl Zeiss, Oberkochen, Germany) and the images were taken using a digital camera (AxioCam MRc5, Carl Zeiss, Oberkochen, Germany).

### 4.5. Immunohistochemistry (IHC) Analysis

Articular cartilage samples were processed for immunohistochemical analysis as previously described [37]. In brief, the sections were de-waxed in xylene, hydrated in graded ethanol scale and incubated in 0.3% H_2_O_2_/PBS to stop endogenous peroxidase activity for 30 min. Afterwards, the slides were cleaned for 20 min with PBS (Bio-Optica, Milan, Italy). The slides were heated in a microwave oven (5min×3, 750W, LG Electronics Italia S.p.A., Milan, Italy) in Tris-EDTA buffer (pH 8.0; Bio-Optica, Milan, Italy) or in citrate buffer–pH 6 (pH 6.0; Bio-Optica, Milan, Italy), for the antigenic retrieval [38]. Afterwards, the slides were incubated overnight at 4 °C with diluted rabbit polyclonal antibodies against types I collagen (ab34710; Abcam, Cambridge, UK) and type II collagen (ab34712; Abcam, Cambridge, UK); rabbit monoclonal anti-SOX9 (ab185966; Abcam, Cambridge, UK) and anti-aggrecan (ab3778; Abcam, Cambridge, UK) antibodies, diluted 1:100 in PBS (Sigma-Aldrich, Milan, Italy). Immune-complexes were then incubated with biotinylated link antibodies (HRP-conjugated anti-rabbit and anti-mouse were used as secondary antibodies) and detected with peroxidase-labelled streptavidin (LSAB + System-HRP, K0690, Dako, Glostrup, Denmark). Immunoreactivity was labelled using 0.1% 3,3′-diaminobenzidine (DAB) (DAB substrate Chromogen System; Dako, Glostrup, Denmark). The Mayer’s hematoxylin (Histolab Products AB, Göteborg, Sweden) was used for the counterstain and then the sections were mounted in GVA (Zymed Laboratories, San Francisco, CA, USA), observed with an Axioplan Zeiss light microscope (Carl Zeiss, Oberkochen, Germany) and captured with a digital camera (AxioCam MRc5, Carl Zeiss, Oberkochen, Germany).

### 4.6. Computerized Morphometric Measurements and Image Analysis

One field of about 550,000 µm^2^, corresponding to the defect area, carefully selected from each section (three sections for each time point), was analysed for histochemical assessment of Alcian Blue staining, detecting GAGs expression, and to quantify the level of positive anti-Collagen I, anti-Collagen II, anti-Aggrecan and anti-SOX9 antibodies immunoexpression. The image analysis software (AxioVision Release 4.8.2-SP2 Software, Carl Zeiss Microscopy GmbH, Jena, Germany), which quantifies the staining level as the densitometric count (pixel^2^) normalized to the defect area of each sample, was used. The samples were analysed by using the Zeiss Axioplan light microscope (Carl Zeiss, Oberkochen, Germany) and the pictures were taken with a digital camera (AxioCam MRc5, Carl Zeiss, Oberkochen, Germany). Two investigators (one anatomical morphologist and one histologist) made the morphological assessment. If disputes occurred, a unanimous agreement was reached after section re-evaluation and before proceeding with data interpretation.

### 4.7. Quantitative Real-Time Polymerase Chain Reaction (q-PCR)

Total RNA was isolated from paraffin-embedded tissue sections by using the RNeasy FFPE Kit (Qiagen, Germantown, MD, USA). cDNA was synthesised from 1 μg of total RNA using a High-Capacity cDNA Reverse Transcription Kit (Applied Biosystems). Quantitative RT-PCR was performed using the SYBR Green method on a 7900HT Real Time PCR (Applied Biosystems).

Specific primers for chondral genes, including *COL1A1, COL2A1, aggrecan* and *SOX9*, were designed using Primer Blast [39] and selecting exon-exon junctions on mRNA as a target region for annealing. Gene expression was assessed using the 2^−ΔΔCt^ method [40]. Oligonucleotide sequences are reported in Table 2. Results were normalised to the levels of Beta-Tubulin (TUBB), used as an endogenous control.

### 4.8. Statistical Analysis

The statistical evaluation was carried out by using GraphPad Instat^®^ Biostatistics version 3.0 software (GraphPad Software, Inc., La Jolla, CA, USA), as previously described [41]. Differences between experimental groups were evaluated by using a two-way ANOVA followed by Tukey’s multiple comparison post hoc test. Datasets were tested for normal distribution with the Kolmogorov–Smirnov test. All variables were normally distributed. For all experiments, *p*-values of less than 0.05 were considered statistically significant (*p < 0.05; **p < 0.01; ***p < 0.001; ****p < 0.0001 and ns, not significant). The data are presented as the mean value ± SD, as previously described [42]. 

## 5. Conclusions

In conclusion, our data support the high biocompatibility of the collagen I-based scaffold, which is able to efficaciously integrate into the host articular cartilage and to promote the development of new cartilage-like tissue by recruiting the host cells and driving them towards the chondrogenic differentiation. Moreover, thanks to the good biodegradability over time (up to 16-weeks), this scaffold represents a promising tool for cartilage tissue engineering and repair approaches.

## Figures and Tables

**Table 1:** Experimental groups.

Study Groups	Time Points	Number of Rats
CTRL	4, 8, 16 weeks	n. 9 (3 × each time point)
ACL (only lesion)	4, 8, 16 weeks	n. 9 (3 × each time point)
ACL-S (lesion + scaffold)	4, 8, 16 weeks	n. 9 (3 × each time point)

**Table 2:** Primer sequences.

Target Gene	Forward	Reverse
*COL1A1*	CCGGAAACAGACAAGCAACCCAAA	AAAGGAGCAGAAAGGGCAGCATTG
*COL2A1*	TGGTCTTGGTGGAAACTTTGCTGC	AGGTTCACCAGGTTCACCAGGATT
*Aggrecan*	TGTGGTGATGATCTGGCACGAGAA	CGGCGGACAAATTAGATGCGGTT
*Sox9*	AACAACCCGTCTACACACAGCTCA	TGGGTAATGCGCTTGGATAGGTCA
*TuBB4a*	GACGTGAGTACTGCTCCGC	CTTGCAGGTGCACGATTTCC
